# Study on morphometry of distal humerus and its clinical correlations

**DOI:** 10.6026/973206300200859

**Published:** 2024-08-31

**Authors:** Shivani Azhagiri, Purvi Venkatesh, Srimathi Ramasamy, Kalpana Ramachandran

**Affiliations:** 1 Department of Anatomy, Sri Ramachandra Institute of Higher Education and Research (DU), Porur, Chennai-600116, Tamilnadu, India

**Keywords:** humerus, elbow joint, arthroplasty, prosthesis design, humeral fractures, distal

## Abstract

Morphometric analysis of bones gives essential information for reconstructive surgeries and prosthetics. It is also useful in forensic medicine to identify and estimate age, sex and height from skeletal remains. We have measured and analyzed the following parameters in 100 distal humerii: the maximal length of the humerus, transverse distance between medial epicondyle and lateral epicondyle, horizontal diameter of trochlea, antero-posterior diameter of trochlea, and horizontal distance from medial epicondyle to capitulum. Distal humeral fractures are often challenging and difficult to treat which requires knowledge of distal humeral anatomy. Data from this study and the comparisons made will help in this.

## Background:

The distal part of the humerus has medial epicondyle, trochlea, capitulum and lateral epicondyle respectively in order from the medial to the lateral side [1]. Understanding the average parameters of long bone segments is particularly beneficial to determine the identity of a skeleton and to create different implants for humeral fracture repair. [2] Therefore, it is of interest to highlight the differences in the measurements in different groups of people and to clinically correlate in the production of prostheses and implants for elbow arthroplasty.

## Materials and Methodology:

A hundred dried adult humeral bones were procured from the Department of Anatomy in Sri Ramachandra Medical College. The bones were divided into right sided and left sided humerii. Inclusion criteria are Intact, non-pathological dried adult humerii. Exclusion criteria - Humerus with any gross deformity or damage or poor condition. An osteometric board and a vernier caliper was used to measure the following parameters: maximal length of the humerus- measured from the tip of the humerus to the apex of the trochlea (using an otseometric board of least count 1mm), transverse distance between medial epicondyle and lateral epicondyle, horizontal diameter of trochlea, anteroposterior diameter of trochlea and horizontal distance from medial epicondyle to capitulum. The measurements of distal humerus were done with a vernier caliper of least count 0.1 mm. The values were recorded in Microsoft Excel. The mean values and the standard deviation were determined for each parameter. SPSS Software was used to analyze the data. The findings of the five parameters measured are recorded as follows.

## Results:

The maximal length was found to be 306.69±22.99. The transverse diameter between medial and lateral epicondyle was found to be 57.23±4.22. The horizontal diameter of trochlea was found to be 24.76±2.38. The anteroposterior diameter of trochlea was found to be 16.65±2.03. The horizontal distance from medial epicondyle to capitulum was found to be 54.63±4.0. (Table 1) The maximum length of the right humeral bones was 309.98±26.58 while the left humeral bones were found to be 303.40±18.41. The distal humeral parameters did not show any significant variation between the right and the left sides. (Table 2)

## Discussion:

The elbow is a compound joint, fracture of which is difficult to treat. In a third of all humeral fractures the distal humerus is fractured. 2% of all fractures in adults are distal humeral fractures. Surgery is the treatment of choice in such cases and conservative treatment is not preferred [4]. Surgical treatment has been successful in the long term with favourable outcomes [5]. Distal Humeral Fractures are most often treated successfully with surgery. Open Reduction and Internal Fixation is most commonly done. In some cases, which are more complicated a Total Elbow Arthroplasty may need to be done. The measurements of the parameters in this study along with various other parameters are required to perform a Total Elbow Arthroplasty. [6] Distal humeral fractures pose an even greater challenge in patients over 65 years as the bone involved is fragile. A third of these patients develop complications. [7] Complications following Total Elbow Arthroplasty have also been reported. Distal Humeral Prosthetic Hemiarthroplasty is also an option for which a thorough understanding of the distal humeral anatomy and the anatomy of proximal radius and ulna is required. [8] Total elbow arthroplasty has also been used as a successful treatment option for a patient with distal humerus osteosarcoma. [9] The humeral bone is also of significance in the forensic sciences for identification. The parameters measured here show variations with ethnicity and region. [1] The maximum length of the humerus is important in determining characteristic features in forensic and anthropological studies. [10] On comparison, the values of the maximal length and the parameters of the distal humerus show minor variations in the studies conducted by Vinay *et al.* Ashiyani *et al.* and Narayana & Himabindu. This shows that there are variations in the distal humeral measurements within the Indian subcontinent itself. These variations must be taken into account while treating a Distal Humeral Fracture. (Table 3)

## Conclusion:

This study measures the maximal length of the humeral bone and the important parameters of the distal humerus in 100 humeral bones in the South Indian population, divided into 50 left sided and 50 right sided bones, and provide the mean values and comparison between right and left humeral bones. These values on comparison with other studies show variations in certain parameters which have implications in the options chosen to treat fractures of the distal humerus as knowledge about the morphometry is essential in any surgical procedure that is chosen. Distal Humeral fractures are difficult to treat, and the values and variations derived in these studies will help clinically in treating them and in designing prostheses and implants in the future.

## Figures and Tables

**Table 1:** Descriptive Statistics of the parameters measured.

	**PARAMETERS**	**MEAN**	**STANDARD DEVIATION**	**MINIMUM VALUE**	**MAXIMUM VALUE**
P1	Maximum Length	306.69	22.99	250	360
P2	Transverse Distance between Medial and Lateral Epicondyle	57.23	4.22	46.7	66.5
P3	Horizontal Diameter of Trochlea	24.76	2.38	18.6	32.2
P4	Anteroposterior Diameter of Trochlea	16.65	2.03	12.3	24.7
P5	Horizontal Distance from Medial Epicondyle to Capitulum	54.63	4.08	46	64

**Table 2:** Comparison of the parameters between left and right sides

	**Parameters**		**Mean**	**Standard Deviation**	**Standard Error Of Mean**
P1	Maximum Length	Left	303.4	18.41	2.6
		Right	309.98	26.58	3.76
P2	Transverse Distance Between Medial and Lateral Epicondyle	Left	56.91	4.07	0.57
		Right	57.55	4.39	0.62
P3	Horizontal Diameter of Trochlea	Left	24.76	2.46	0.35
		Right	24.76	2.34	0.33
P4	Anteroposterior Diameter of Trochlea	Left	16.13	2.05	0.29
		Right	17.16	1.89	0.27
P5	Horizontal Distance from Medial Epicondyle to Capitulum	Left	54.07	3.92	0.55
		Right	55.18	4.19	0.59

**Table 3:** Comparison with different studies

	**Parameters**		**Vinay *et al.*[1]**	**Ashiyani *et al.*[2]**	**Siva Narayana and Himabindu [3]**	**Present study**
P1	Maximum Length	Left	301.13±22.44	303.2±15.8	-	303.40±18.41
		Right	306.32±21.98	303.9±16.6	-	309.98±26.58
P2	Transverse Distance Between Medial and Lateral Epicondyle	Left	56.02±4.77	55.8±4.2	57.0±4.6	56.91±4.07
		Right	57.40±4.82	56.6±3.6	58.0±4.0	57.55±4.39
P3	Horizontal Diameter of Trochlea	Left	23.57±2.61	22.4±2.0	22.4±2.2	24.76±2.46
		Right	24.43±2.69	22.6±1.8	22.4±2.2	24.76±2.34
P4	Anteroposterior Diameter of Trochlea	Left	16.35±3.77	14.5±1.7	15.6±1.8	16.13±2.05
		Right	17.05±3.96	14.5±1.5	15.6±1.8	17.16±1.89
P5	Horizontal Distance from Medial Epicondyle to Capitulum	Left	52.68±6.63	53.9±4.1	56.0±4.5	54.07±3.92
		Right	54.56±4.9	54.2±3.3	56.3±3.7	55.18±4.19
